# Proliferative proteome induced by HPV 16E6 and NFX1-123 partnership in longitudinal keratinocyte cultures

**DOI:** 10.1128/jvi.00967-25

**Published:** 2025-08-13

**Authors:** C. L. Billingsley, E. H. Doud, W. Smith-Kinnaman, A. L. Mosley, S. Chintala, R. A. Katzenellenbogen

**Affiliations:** 1 Department of Microbiology and Immunology, Indiana University School of Medicine 734638 , Indianapolis, Indiana, USA; 2 Biochemistry and Molecular Biology, Indiana University School of Medicine, Indianapolis 207034 , Indianapolis, Indiana, USA; 3 Center for Proteome Analysis, Indiana University School of Medicine 12250, Indianapolis, Indiana, USA; 4 Center for Computational Biology and Bioinformatics, Indiana University School of Medicine 12250, Indianapolis, Indiana, USA; 5 Department of Pediatrics, Indiana University School of Medicine 145785, Indianapolis, Indiana, USA; Dartmouth College Geisel School of Medicine 12285, Hanover, New Hampshire, USA

**Keywords:** human papillomavirus (HPV), E6, NFX1-123, cervical cancer

## Abstract

Human papillomaviruses (HPVs) cause cervical, anogenital, and oropharyngeal cancers. Despite the availability of preventive HPV vaccines, their poor uptake leaves individuals at risk for these cancers, many of which remain common globally and others that are increasing domestically. The HPV 16 E6 (16E6) protein plays a significant role in inducing and maintaining cellular transformation of its infected host cell; however, 16E6 itself has no enzymatic activity and executes its functions through partnerships with host proteins. Previously, we demonstrated that 16E6 binds to the host protein NFX1-123, and NFX1-123 expression is increased in HPV 16-positive cervical cancer cell lines and primary cancers compared to normal tissues. In this study, we quantify growth patterns of 16E6-expressing keratinocytes with endogenous or overexpressed NFX1-123 (16E6/vec and 16E6/FN123, respectively) levels and interrogate proteome expression changes early and late in longitudinal growth cultures. Early-passage 16E6/vec and 16E6/FN123 cells showed similar growth rates; however, late-passage 16E6/FN123 cells had accelerated growth, greater population doublings, increased telomerase activity, and a dysplastic phenotype when compared to 16E6/vec cells. Matching this, mass spectrometry revealed only 22 differentially expressed proteins at early passage; meanwhile, late-passaged cells had 827 differentially expressed proteins among 16E6/FN123 and 16E6/vec cells. In that, DNA maintenance and repair, proteins, namely, MCM2 and MCM4, were highly expressed in the 16E6/FN123 compared to 16E6/vec cells. These findings indicate that the 16E6 and NFX1-123 partnership, especially with sustained higher levels of NFX1-123, alters the longitudinal cellular environment in a manner that may initiate a preneoplastic phenotype.

**IMPORTANCE:**

The high-risk HPV 16E6 protein plays a significant role in inducing and maintaining cellular transformation of its infected host cell; however, 16E6 has no enzymatic activity and carries out most of its functions through partnerships with host endogenous proteins, one being NFX1-123. That NFX1-123 expression is increased in HPV 16-positive cervical cancer and primary cancers compared to normal tissues, and NFX1-123 binds directly to 16E6. This study leverages proteomics to reinforce the synergistic actions of HPV16E6 and NFX1-123 and identifies cellular pathways impacted by this protein partnership over time. These findings encourage further investigation of NFX1-123 as a potential therapeutic target of HPV 16-positive tissues.

## INTRODUCTION

Human papillomaviruses (HPVs) are small, non-enveloped, double-stranded DNA viruses that are part of the Papillomaviridae family. To date, there are over 200 types of HPVs that have been identified (1, 2), a subset of which are considered high-risk (HR) HPV types, which have a high risk of causing cancer. HR HPVs are the causative agent of nearly all cases of cervical cancer (3), are responsible for other anogenital and oropharyngeal cancers (3, 4), and cause 4.5% of cancers worldwide (5). Among HR HPVs, there are common and unique genes and pathways that are activated to drive cancer development, meriting detailed and individualized evaluation. HPV 16 is the most common HR HPV, found in half of cervical cancers globally, so our studies seek to understand how this HR HPV alters its infected host to create an environment conducive for cancer development.

In HPV 16, E6 and E7 are two viral oncoproteins that create a permissive cellular environment for viral replication, in addition to their roles in cancer development. Normally, keratinocytes in the basal layer of stratified squamous epithelium are the only cell type that can grow, replicate DNA, and divide. While keratinocytes stop dividing and begin to differentiate in the suprabasalar and upper layers of stratified squamous epithelium, here HPV 16 E6 (16E6) and E7 (16E7) support continued host cell DNA replication, cellular growth, and cellular division (6). To immortalize infected epithelia, both 16E6 and 16E7 must be expressed.

For 16E6, it affects the host cell through cellular protein partnerships. One key partner for 16E6 is the E3 ubiquitin ligase E6-associated protein (E6AP). 16E6 and E6AP polyubiquitinate p53 and target it for proteasomal degradation (7, 8), permitting dysregulated growth of HPV-infected keratinocytes by blocking apoptosis and allowing more efficient viral genome replication during the maintenance and amplification stages of the HPV life cycle. Loss of apoptotic signaling is also a key step in cancer development (9–11).

In addition to their ability to degrade p53, 16E6 and E6AP also push cells toward immortalization by activating and augmenting telomerase activity during a long-term HPV 16 infection (12–14). Telomerase extends guanine-rich repetitive sequences (TTAGGG) of telomeric DNA at the ends of linear chromosomes (15). In normal somatic diploid cells, telomerase is not active, so telomeric DNA shortens after each cellular division, marking the age of a cell (16). However, if telomerase is active, telomeric DNA is extended during DNA replication; this allows cells to avoid senescence, which is required for cellular immortalization. All HPV-associated cancers have telomerase activity (17), and when this activity is reduced *in vitro*, cells no longer grow in culture (18).

Published studies have demonstrated that 16E6 itself binds directly to and affects another cellular protein, NFX1-123 (18, 19). A tandem affinity purification assay showed that NFX1-123 bound USP9X (also known as FAF-X), a ubiquitin-specific protease that can remove ubiquitin moieties from proteins (19). Additional studies showed that 16E6 increased USP9X, leading to reduced NFX1-123 protein ubiquitination and increased total protein levels of NFX1-123 (20). NFX1-123 abundance is increased in HPV 16-positive cervical cancer cell lines, in HPV 16-positive cervical dysplasia, and in primary cervical cancers compared to normal tissues (21); NFX1-123’s high expression is important for full telomerase activity and cellular growth. When NFX1-123 expression is reduced, cervical cancer cell line growth slows; telomerase activity is blunted; colony formation is decreased; and cell migration and wound healing are reduced (21, 22).

Typically, there are more than 10 years between an initial HR HPV infection and cervical cancer development (3), but to date, it is unclear how higher expression of NFX1-123, in partnership with 16E6 over time, may drive differences in gene expression and cellular pathways that initiate oncogenesis. Here, we quantified growth and phenotypic differences in 16E6-expressing cells with greater or typical levels of NFX1-123 and then performed quantitative proteomics to identify and study proteins and pathways that change. Parsing the role of NFX1-123’s direct protein partner 16E6 from 16E7 or other HPV genes and proteins, we leverage proteomics to identify how the early and sustained modulation of NFX1-123 in a host cell can affect key oncogenic genes and pathways. Of the top 25 upregulated pathways, we identified 3 telomeric regulatory pathways that were amplified concurrent with higher levels of NFX1-123 over time. We also measured 43 mitotic proteins commonly increased at the RNA level in cervical cancers, which included MCM2 and MCM4, that were elevated with 16E6 and high NFX1-123. These findings reveal changes in protein homeostasis that occurred because of 16E6 and greater NFX1-123.

## RESULTS

### Greater NFX1-123 increased population doublings of 16E6 cells

**Fig 1:**
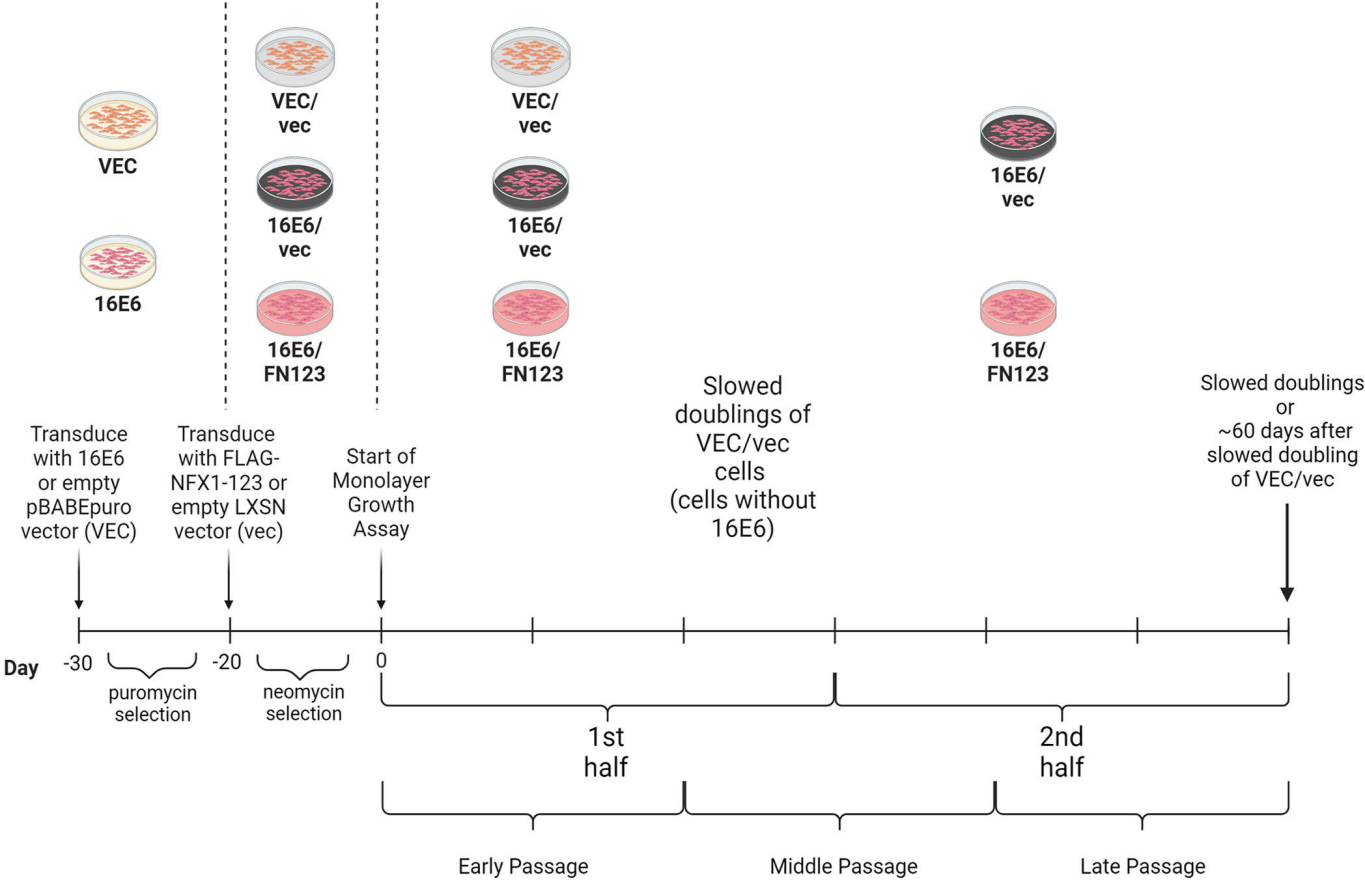
Graphical timeline of the monolayer HFK model. Three biologically unique HFK cell lines were transduced with 16E6 or a pBABE control (VEC), then transduced with either FLAG-tagged NFX1-123 (FN123) or an LXSN control (vec). Cells were plated at a density of 5 × 10^5^ cells/plate and then grown for 3 days. On day 3, cells were counted and replated at a density of 5 × 10^5^ cells/plate. This continued until cells experienced slowed population doublings, which we defined as a population doubling less than 2 in 3 days, or until 60 days after the slowed doubling of the VEC/vec cells of the respective biological background. Figure created with Biorender.com.

To quantify the long-term impact of the 16E6 and NFX1-123 partnership and the role that increased NFX1-123 has in driving gene and biological pathway changes in disease, primary human foreskin keratinocytes (HFKs) with 16E6 and endogenous or overexpressed NFX1-123 were grown in culture, and population doublings were measured to elucidate the growth parameters of the cell lines (Fig. 1). To establish rigor and mimic the variability that can occur with the natural infection process from person to person, three biologically independent HFK cell lines (HFK-A, HFK-B, and HFK-C) were used.

**Fig 2:**
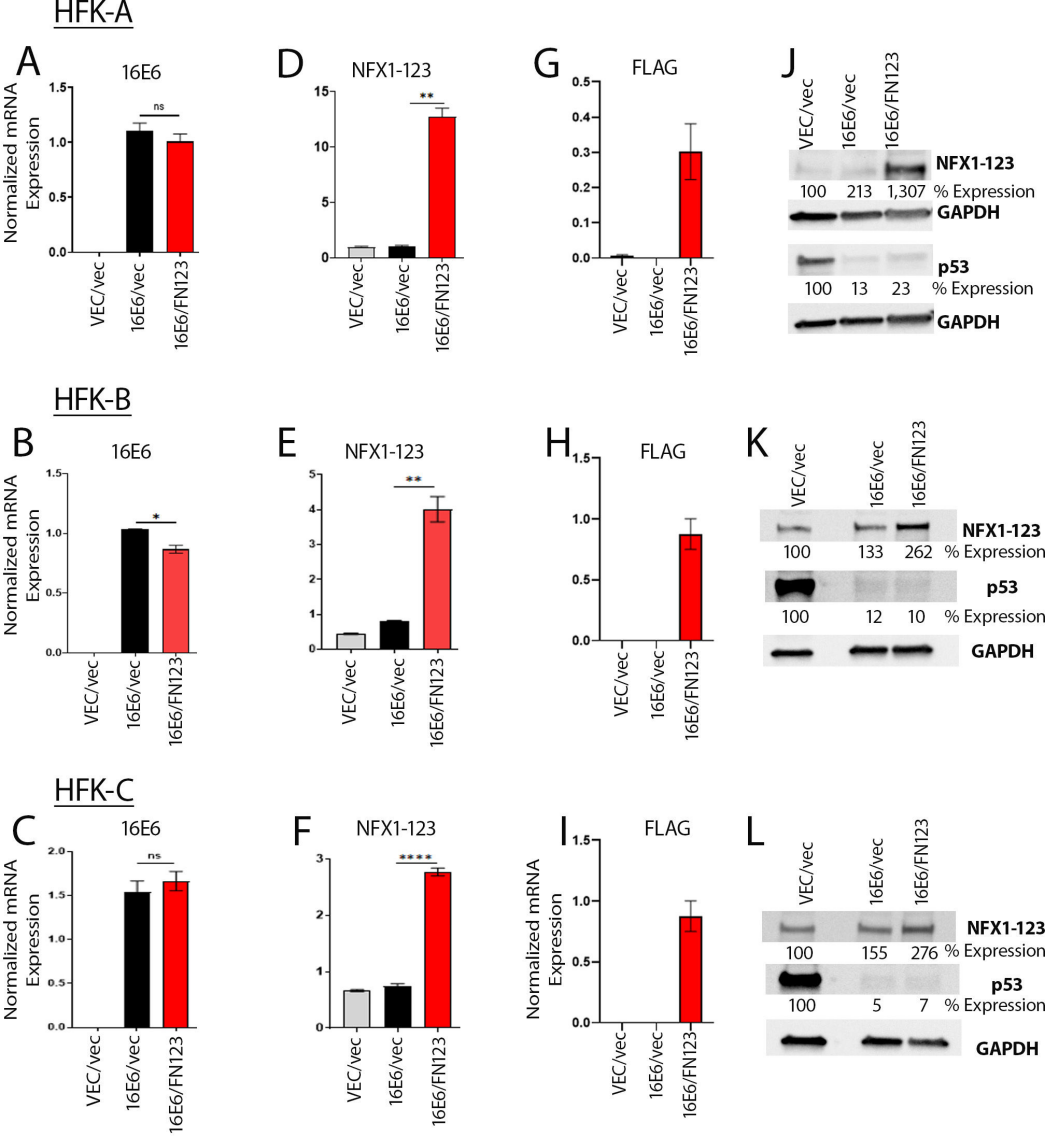
Validation of HFK-A, HFK-B, and HFK-C lines generated in growth assay. (**A–C**) 16E6, (**D–F**) NFX1-123, and (**G–I**) FLAG-tagged NFX1-123 mRNA expression was measured by reverse transcription quantitative PCR and normalized to 36B4. (**J–L**) Immunoblotting of whole cell protein extracts confirms greater NFX1-123 in FN123 and reduced p53 levels in 16E6 cell lines. Densitometry of proteins (% expression) normalized to GAPDH and VEC/vec expression listed. Error bars represent the standard deviation of three technical replicates. *, *P* < 0.05; **, *P* < 0.005; ****, *P* < 0.0005. ns, non-significant.

To confirm 16E6 expression and overexpression of FLAG-tagged NFX1-123 at both mRNA and protein levels after serial transduction and selection, we conducted quantitative PCR and Western blot studies, respectively. In all 16E6 HFK cells, 16E6 mRNA was detected (Fig. 2A through C) and p53 was reduced (Fig. 2J through L). FLAG-tagged NFX1-123 mRNA was detected in the HFK cells with overexpressed NFX1-123 (Fig. 2D through F), indicating the presence of exogenous NFX1-123 in these cells. NFX1-123 mRNA was expressed in all cells as it is an endogenously expressed gene in HFKs, but significantly higher levels of NFX1-123 in the HFK cells with overexpressed NFX1-123 were seen (Fig. 2G through I). Higher NFX1-123 protein levels were also quantified in the HFK cells with overexpressed NFX1-123 (Fig. 2J through L). Taken together, these findings validated the genetic changes introduced within the three HFK cell lines.

**Fig 3:**
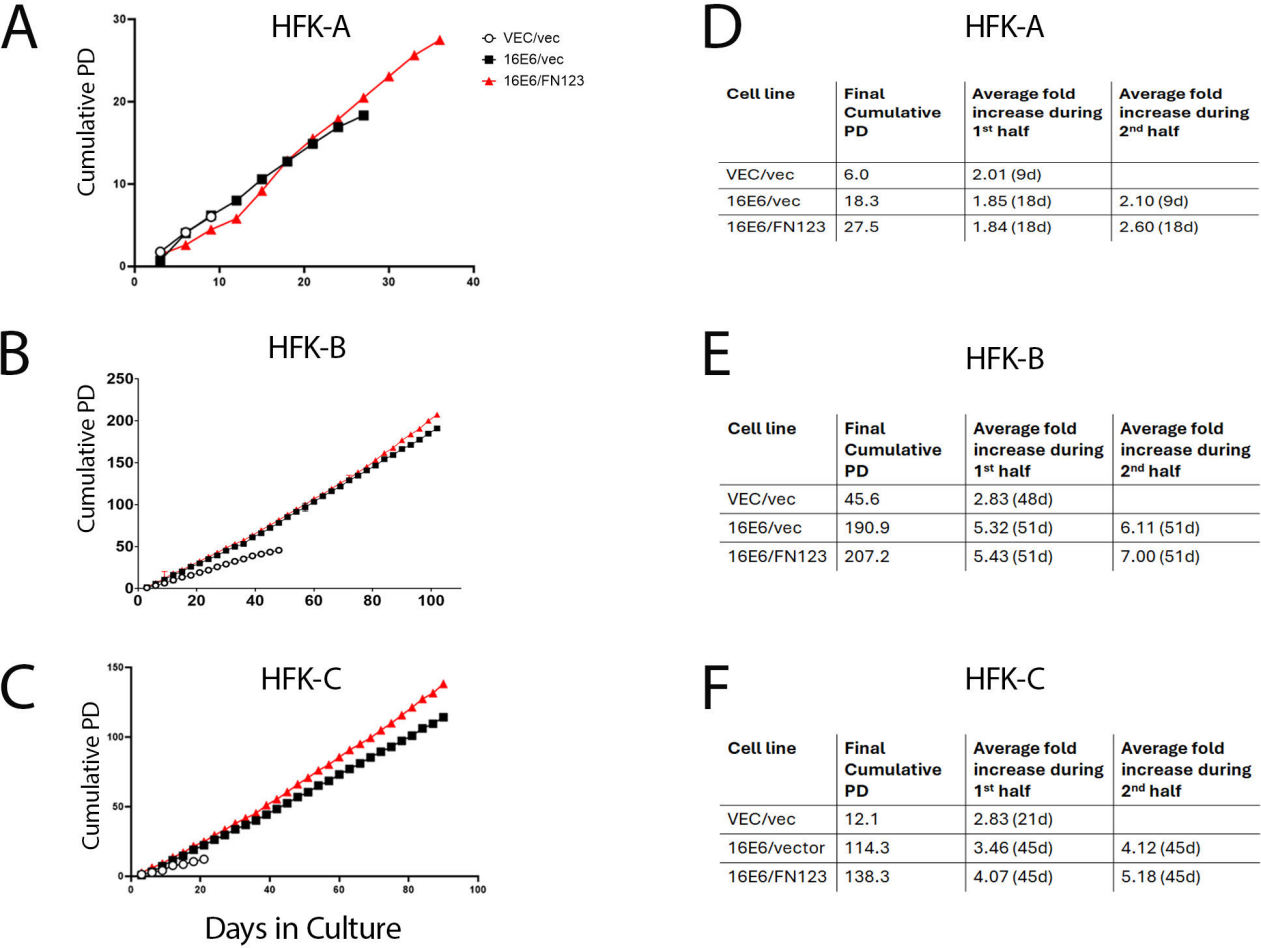
NFX1-123 increased population doublings of 16E6 HFK-A, HFK-B, and HFK-C. (**A–C**) Cumulative population doublings for the VEC/vec (open circle), 16E6/vec (black square), and 16E6/FN123 (red triangle) for the HFK-A, HFK-B, and HFK-C cell lines. (**D–F**) Average fold change was calculated by dividing the cumulative population doubling over a set number of days (listed in parentheses) to obtain growth characteristics for the 1st half vs 2nd half of the assay.

Across these three biologically unique HFK cell lines, cells without 16E6 consistently experienced fewer population doublings than HFKs with 16E6 (Fig. 3B through D). This is supported by previous literature that the HR E6 protein extends cellular growth over time and permits transformation of epithelial cells (7, 8, 12, 13, 23, 24). We overexpressed NFX1-123 in HFKs without 16E6 in each of these biological replicates, and these cells closely resembled the growth pattern of the VEC/vec HFKs (data not shown). This indicated that NFX1-123 alone was not responsible for augmented population doublings. At matched time points, the 16E6/FN123 HFKs consistently showed greater proliferation than 16E6 HFKs with endogenous expression of NFX1-123, as demonstrated by higher population doublings and final cumulative population doublings (Fig. 3A through C). To further determine the differences in growth pattern across the different HFK cells over time, we calculated the average fold change of growth in the first half compared to the last half of longitudinal growth. In the last half, all 16E6/FN123 HFK cells had augmented fold increases in population doublings than the 16E6/vec HFK cells (Fig. 3D through F). These cell lines mirrored our previous work showing that overexpression of NFX1-123 increased the daily and longitudinal proliferation of 16E6-expressing keratinocytes in a non-static or non-linear way (21). Over time, greater NFX1-123 from exogenous expression early in culture led to an acceleration and divergence in growth. This occurred across biologically different HFK backgrounds with distinctly unique abilities to maintain active growth, as HFK-A growth slowed by days 30–40. In contrast, HFK-B and HFK-C maintained the growth of both 16E6/vec and 16E6/FN123 for up to nearly 100 days. Across these distinct HFK biological backgrounds, 16E6/FN123 cells grew well, with either a longer (HFK-A) or more dramatic increase (HFK-B and HFK-C) in cellular doublings with time.

**Fig 4:**
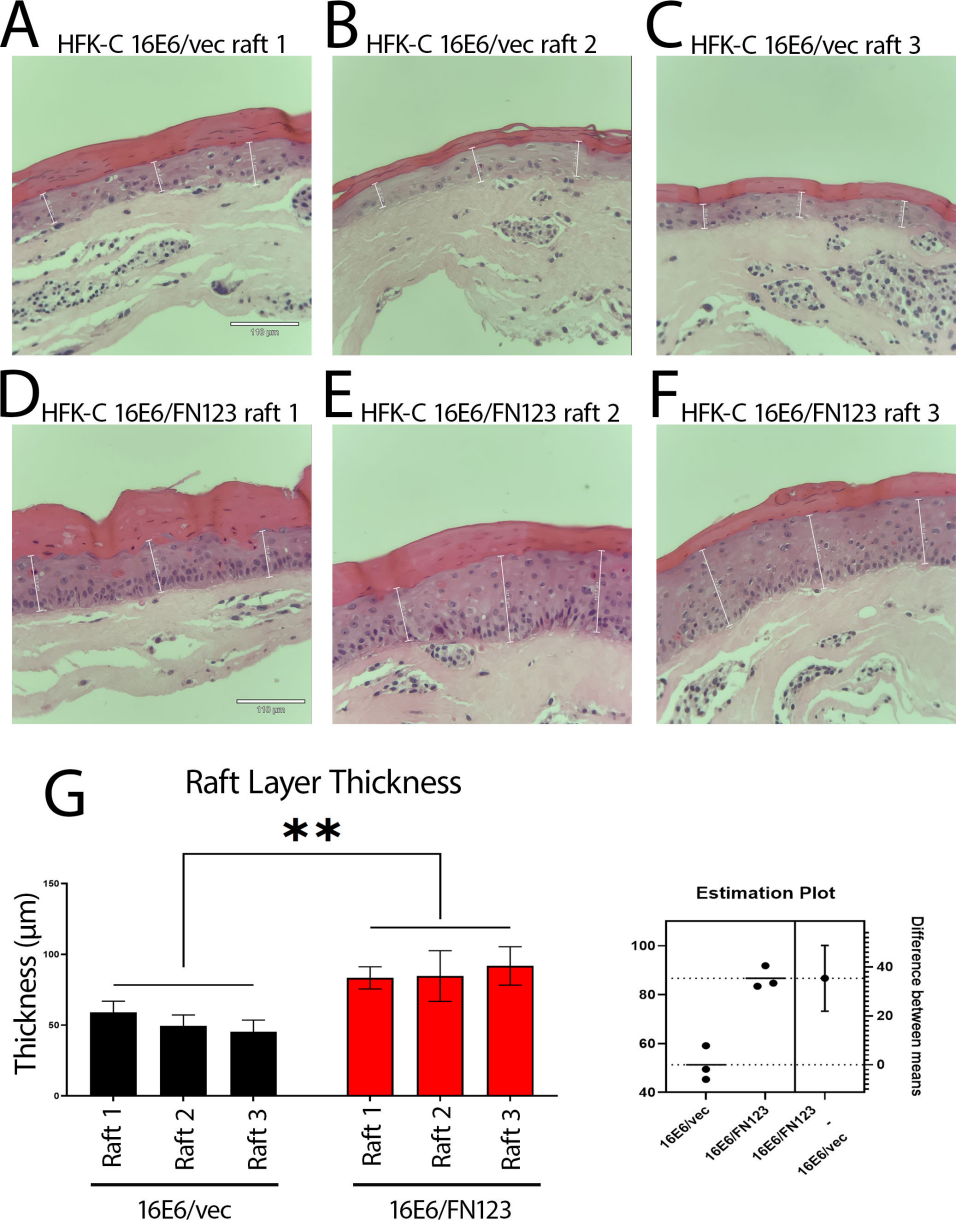
Stratified growth differences are seen in organotypic rafts of late 16E6/FN123 vs late 16E6/vec HFK-C cells. Three technical replicates of 16E6/vec (**A–C**) and 16E6/FN123 (**D–F**) cells were cultured, and images were taken at ×20 magnification on the Revolve microscope. Thickness was measured by calculating the distance from the non-cornified to basal to the start of the cornified layer, as reported on the Revolve microscope. Six fields of view of each raft were measured in three separate areas to generate standard deviations (*n* = 18) (**G**). Statistical significance of all measurements (G, left graph) and the estimated means (G, right graph) was determined using an unpaired *t*-test. **, *P* < 0.005

Monolayer studies are limited as they only represent cellular growth in two dimensions; however, keratinocytes infected by HPV grow as a three-dimensional, stratified squamous epithelium. To mimic this, we generated organotypic raft cultures of the late HFK-C 16E6/vec and late HFK-C 16E6/FN123 cells to elucidate if any growth or phenotypic differences would be seen in the stratified epithelium. Consistently, the 16E6/FN123 HFK-C rafts displayed more nucleated cells along the collagen membrane and demonstrated more dysplastic changes in the 16E6/FN123 cells compared to 16E6/LXSN (Fig. 4). These changes included thickened layers of 16E6/FN123 rafts (Fig. 4G) with greater numbers of nucleated cells in suprabasal layers, mirroring what is seen clinically in cervical intraepithelial lesions, when compared to 16E6/vec rafts.

### Proteome homeostasis changes in late 16E6/FN123 cells vs late 16E6/vec cells

**Fig 5:**
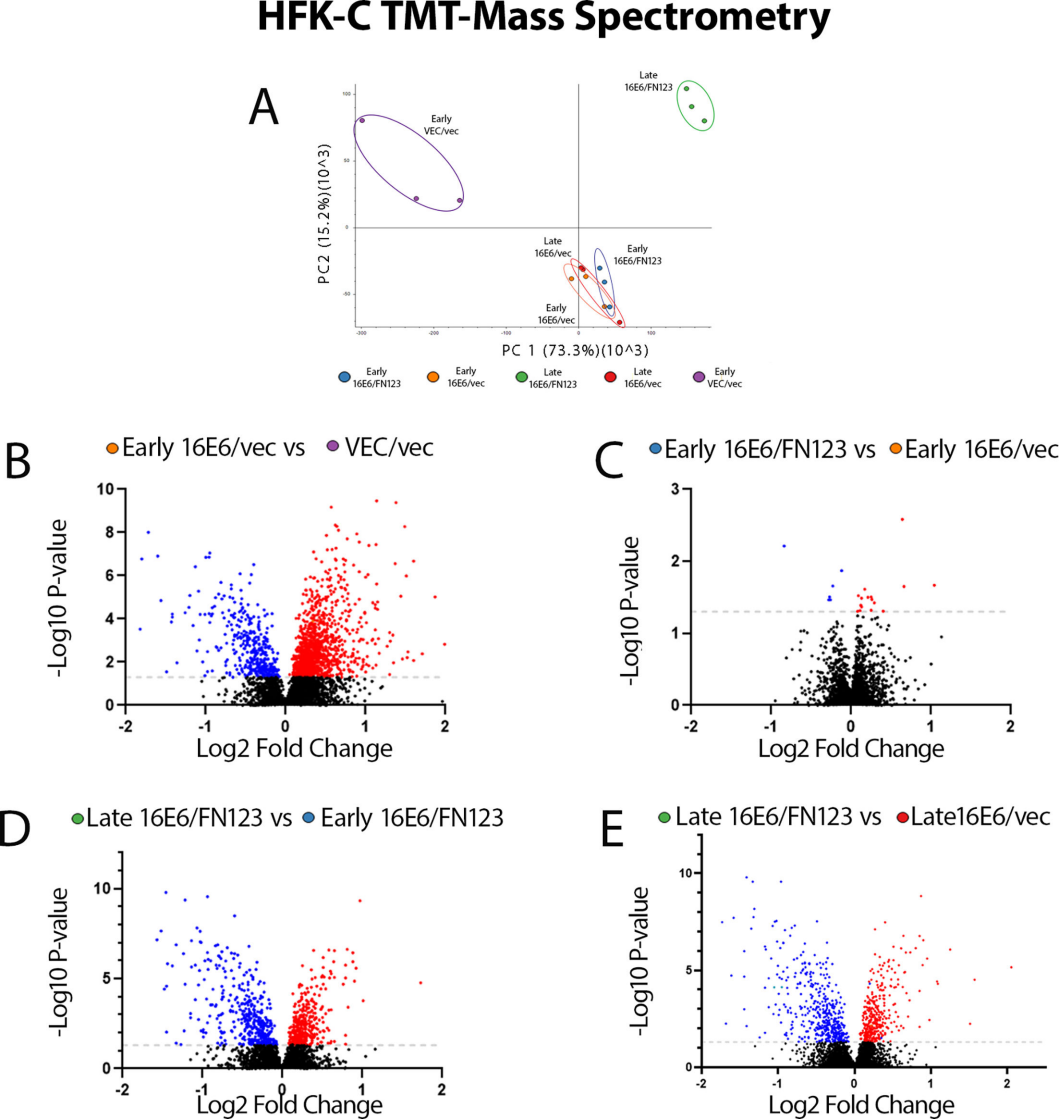
Volcano plots of differentially expressed proteins. Mass spectrometry analysis was performed using protein lysates collected at early and late passages of the growth assay. PCA was used to show the variance of the triplicate samples of each cell line (**A**). Using a *P* value of <0.05 (gray dotted line, −log10 *P* value of >1.3), upregulated proteins, which were identified by having a log2 fold change of >0, are highlighted in red, and downregulated proteins with a log2 fold change of <0 are highlighted in blue (**B–E**). Early 16E6/vec vs VEC/vec (**B**), early 16E6/FN123 vs early 16E6/vec (**C**), late 16E6/FN123 vs early 16E6/FN123 (**D**), and late 16E6/FN123 vs late 16E6/vec (**E**) comparisons. Differential expressions were relative to the first cell line list in each comparison.

Because these longitudinal assays showed phenotypic monolayer and raft growth differences among HFK cells starting with different levels of NFX1-123, we hypothesized that different cellular genes and pathways were activated by the 16E6 and NFX1-123 partnership to promote cellular proliferation at early passages and more robustly at late passages. Based on our previously published studies, we wanted to confirm that telomerase and telomeric DNA regulation would be necessary to these growth differences. Still, we also wanted to extend those data to objectively determine other gene and pathway changes that underpinned growth differences. Because growth changes were most striking in HFK-C, we conducted tandem mass-tagged (TMT) mass spectrometry to perform global protein quantification of the early and late passages of the HFK-C cells. Triplicate biological replicates of each cell line grouped well using principal component analysis (PCA) of the normalized protein abundances (Fig. 5A). The PCA also demonstrated the highest variance between the VEC/vec and the late-passage 16E6/FN123 cells, corresponding with their greatest comparative difference in cumulative population doublings achieved and phenotypic differences in raft cultures.

Significant differential protein abundance was defined as a *P* value less than 0.05 as determined by one-way analysis of variance (ANOVA) statistical tests. The addition of 16E6 to HFK-C cells significantly altered the proteome (1,176 upregulated and 384 downregulated) when compared to VEC/vec (Fig. 5B). Similar significant proteome alterations were seen when comparing early 16E6/FN123 (1,415 upregulated and 453 downregulated), late 16E6/vec (1,481 upregulated and 477 downregulated), and late 16E6/FN123 (1,618 upregulated and 765 downregulated) cells to VEC/vec cells (data not shown). When comparing the early passages of the 16E6/vec and 16E6/FN123 HFK-C cells, there were minimal proteomic changes (16 upregulated and 6 downregulated) (Fig. 5C), which corresponded to the similar population doublings achieved in early passages. In contrast, when comparing the proteomes of the early and late 16E6/FN123 HFK-C cells, there were many more differentially expressed proteins (355 upregulated and 414 downregulated, Fig. 5D), and similar findings were seen when comparing the early and late 16E6/vec cells (106 upregulated and 101 downregulated, data not shown). These dramatic changes in protein expression profiles for late-passage HFK-C cells, both with 16E6 but even more so with overexpressed NFX1-123, were associated with their divergent growth phenotypes and trajectories (363 upregulated and 464 downregulated, Fig. 5E). This indicated a unique proteomic landscape induced by the 16E6 and NFX1-123 partnership over time; it was not a static, tonic state maintained from the initial overexpression of NFX1-123 with 16E6.

### Increased telomere maintenance in late-passage 16E6/FN123 compared to late-passage 16E6/vec cells

**Fig 6:**
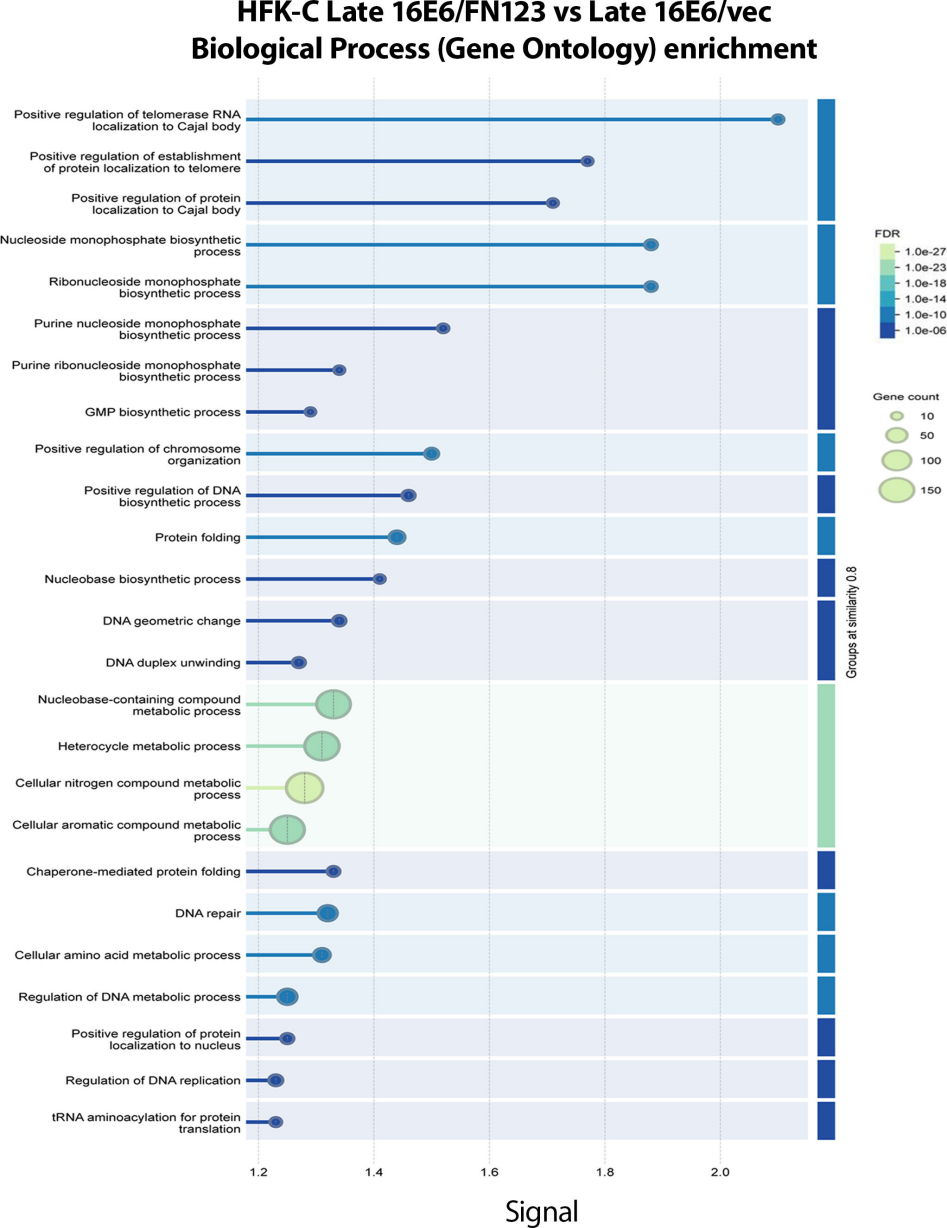
*In silico *(STRING-DB) pathway analysis of protein changes in late 16E6/FN123 vs late 16E6/vec. STRING pathway analysis of the significantly upregulated proteins (*P* value < 0.05, log2 >0) from the late 16E6/FN123 vs late 16E6/vec revealed the top 25 GO biological process pathways. The signal of each pathway is a weighted measure between the observed/expected ratio and the −log(FDR). Pathways were grouped by the similarity in overlapping proteins. The size of the circles corresponded to the number of proteins and subsequent genes that were upregulated in the mass spectrometry data that were associated with the respective pathway.

**Fig 7:**
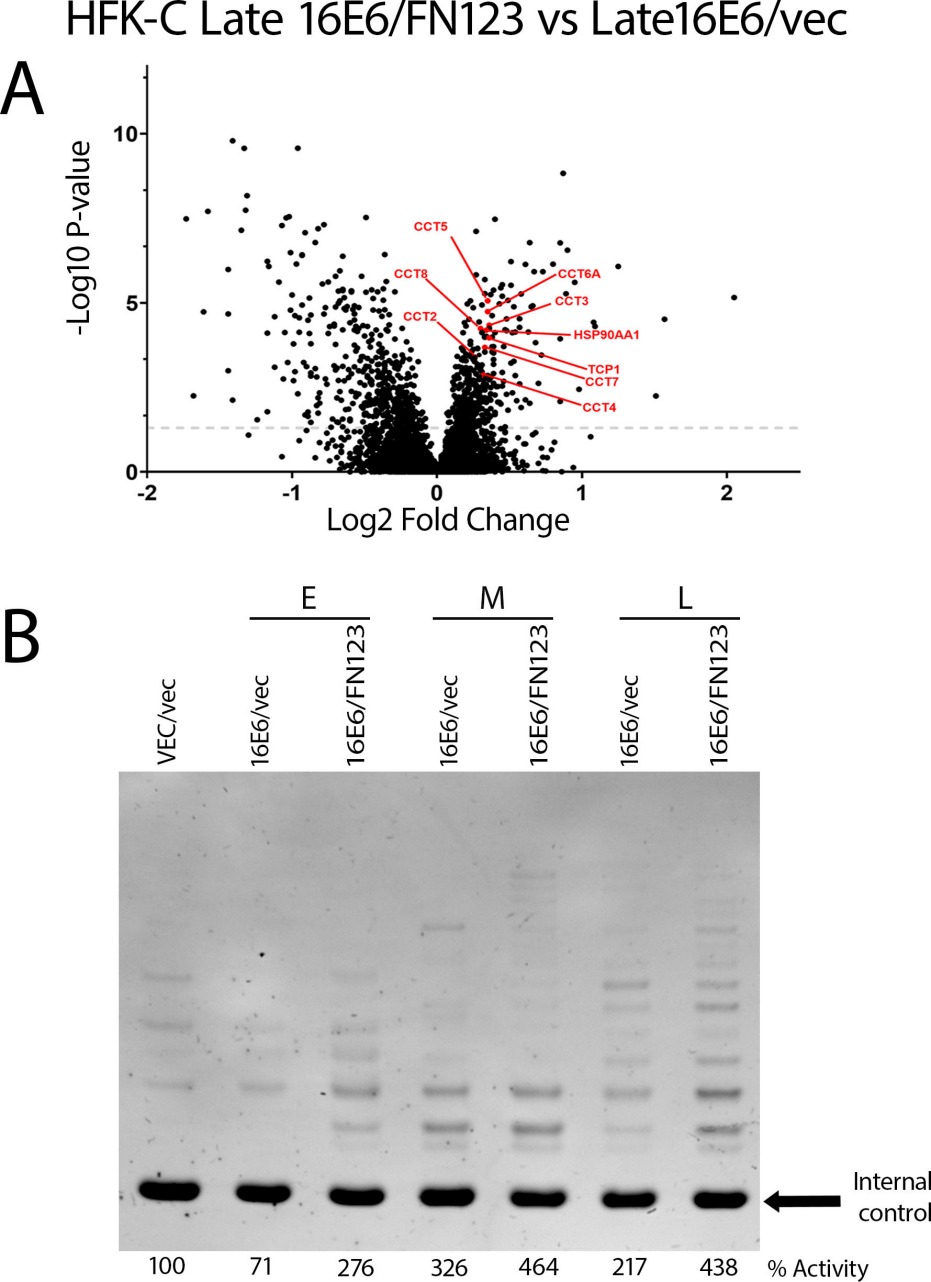
Greater telomerase activity in 16E6/FN123 cells increased over time. Increased abundance of positive telomerase regulation proteins seen on volcano plots of late 16E6/FN123 vs late 16E6/vec (**A**). Cell pellets of the HFK-C cells at early, middle, and late passages were utilized to measure telomerase activity in an* in vitro* assay. Percent expression was normalized to the VEC/vec cells (**B**).

We next sought to understand which cellular pathways were being upregulated or activated in the 16E6 HFK-C cells with overexpressed NFX1-123. *In silico* analysis using STRING-DB (25) of the proteins upregulated in late 16E6/FN123 compared to late 16E6/vec HFK-C cells revealed many cellular pathways as being enriched (Fig. 6). Interestingly, there was an increase in positive telomere regulation pathways. We have previously published that 16E6 and NFX1-123 synergistically increase hTERT, the catalytic subunit of the telomerase enzyme (19, 21); however, this analysis identified that proteins known to be required for proper hTERT activity were also increased. HSP90 and TRiC/CCT complex proteins (CCT1-8) were increased in late 16E6/FN123 compared to late 16E6/vec HFK-C cells (Fig. 7A). While 16E6 is known to directly activate hTERT expression, these proteins, as a collective, optimize hTERT and telomerase protein localization and function (26–33).

We wanted to identify if these higher levels of CCT and HSP90 proteins in the late-passage 16E6/FN123 cells paralleled increased telomerase activity. We compared telomerase activity at early, middle, and late passages of the HFK-C 16E6/vec and 16E6/FN1-123. Similar to our previously published work, we found that telomerase activity was augmented over time in the 16E6/FN123 vs 16E6/vec cells (Fig. 7B). This supports a new foundation and extension for additional proteins in telomerase and telomere-related pathways being influenced by the 16E6/NFX1-123 protein partnership over time, all of which contribute to augmentation of cellular growth and longevity.

### Increased DNA repair pathway proteins in late-passage 16E6/FN123 compared to late-passage 16E6/vec cells

Along with telomere-related pathways being enriched in Gene Ontology biological processes, pathway analysis of cellular proteomic profiles revealed increases in DNA replication and DNA repair pathways, which are indicative of the higher proliferative demands in the late 16E6/FN123 vs late 16E6/vec HFK-C cells (Fig. 6). Collectively, these pathways, telomere and DNA regulation, were increased in the late 16E6/FN123 cell line compared to the late 16E6/vec HFK-C line, indicating a proliferative proteome was induced by 16E6 and greater NFX1-123 over time.

**Fig 8:**
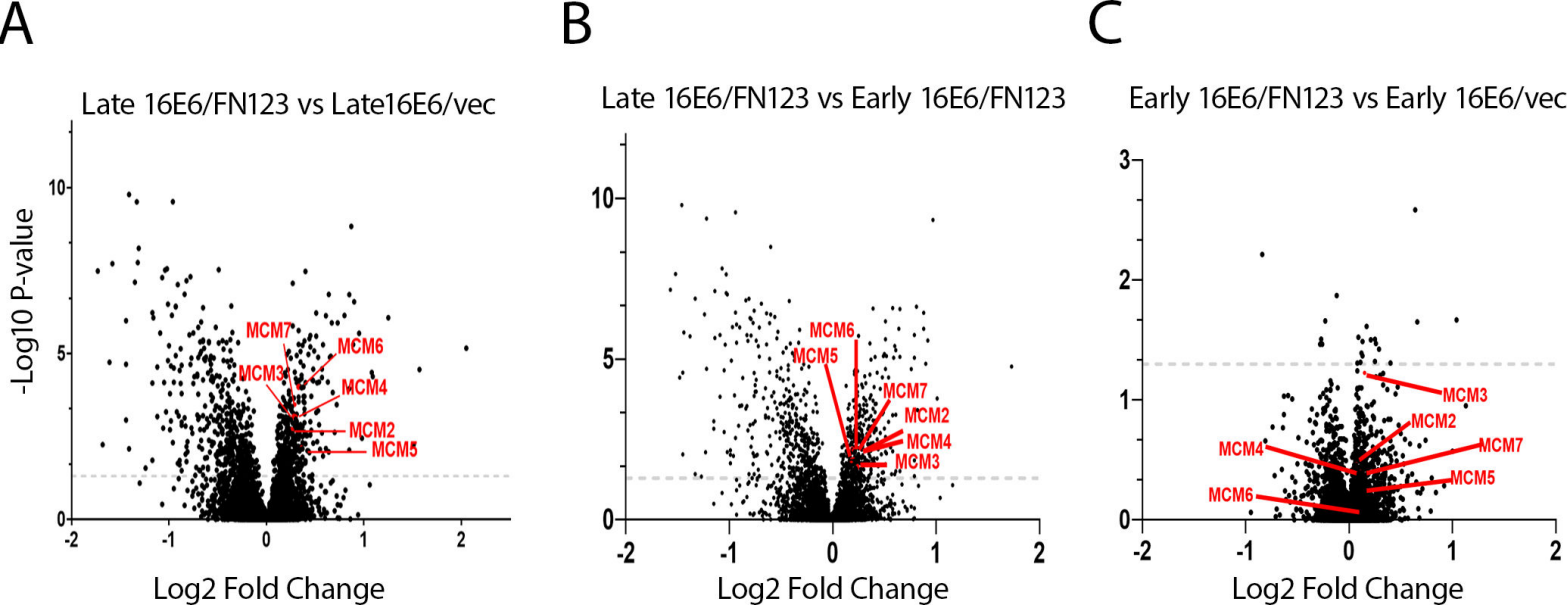
Greater expression of mini-chromosomal maintenance (MCM) proteins involved in DNA repair and DNA metabolic process and replication pathways in HFK-C cell lines. Increased abundance of MCM proteins seen on volcano plots of late 16E6/FN123 vs late 16E6/vec (**A**), late 16E6/FN123 vs early 16E6/FN123 (**B**), but not early 16E6/FN123 vs early 16E6/vec (**C**) HFK-C cell lines. The gray dotted line indicates a *P* value of <0.05.

To specify the unique upregulation of these proteins in the proteome profiles visually, we compared by volcano plot the protein expression of members of the mini-chromosomal maintenance (MCM) family that act in DNA repair, regulation of DNA metabolic process, and DNA replication. In the late 16E6/FN123 to the late 16E6/vec (Fig. 8A) comparison, these MCM family members (MCM2-7) were significantly increased in late 16E6/FN123 cells. These same six MCM proteins were also significantly increased in late 16E6/FN123 cells when compared to early 16E6/FN123 cells (Fig. 8B). These changes were not seen when comparing only early passage 16E6/FN123 and 16E6/vec cells (Fig. 8C), so these protein changes only occurred over time in the cells that started with and maintained augmented NFX1-123 expression. Collectively, this supports the idea that a unique proteome shift is induced by the persistent overexpression of NFX1-123 in 16E6 cells.

### Proteomic temporal trends across HFK biological backgrounds

**Fig 9:**
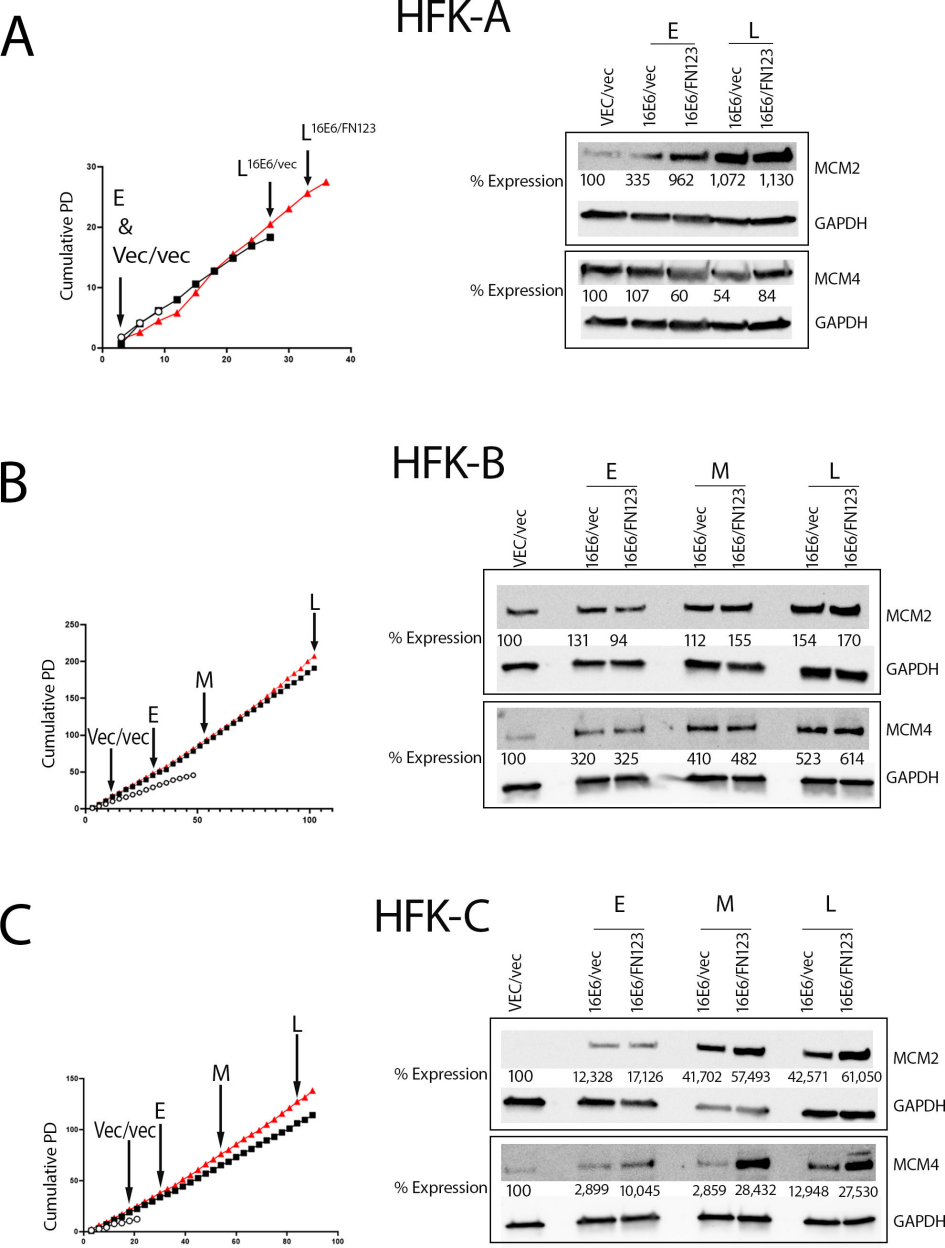
MCM2 and MCM4 increased in HFK-A, HFK-B, and HFK-C cell lines with 16E6/FN123 at late time points vs VEC/vec. MCM2 and MCM4 immunoblotting of VEC/vec, early (labeled E), middle (labeled M), and late (labeled L) 16E6/vec and 16E6/FN123 cell lysates of HFK-A (**A**), HFK-B (**B**), and HFK-C (**C**) densitometry normalized to GAPDH and VEC/vec expression listed.

Because HFK-C was a unique primary cell line, we employed Western blot analysis to discover whether MCM protein increases were universal across the other biological HFK backgrounds. To directly interrogate the mass spectrometry findings of increased MCM2 and MCM4 in late 16E6/FN123 HFK-C, we quantified the expression levels of MCM2 and MCM4 by Western blot at early, middle, and late passages of HFK-B and HFK-C (Fig. 9A through C) and early and late passages of the HFK-A cell line due to its shorter time in culture. MCM2 expression was highest in the late 16E6/FN123 cells with 11.3-, 1.7-, and 610-fold increases in protein compared to the VEC/vec cells in HFK-A, HFK-B, and HFK-C, respectively (Fig. 9A through C, top Western blots). Despite heterogeneity in total growth potential of the HFK-A, HFK-B, and HFK-C cell lines, each biological background had a rise in MCM2 expression as 16E6/FN123 cells were grown in culture, indicating a progressive influence of greater NFX1-123 on MCM2 in 16E6-expressing cells. MCM4 expression was also highest in the late 16E6/FN123 cells with 6.1- and 275.3-fold increases compared to the VEC/vec cells in the HFK-B and C cells, respectively (Fig. 9B and C, bottom Western blots). Similar to MCM2, MCM4 expression gradually increased (from early to middle to late passages), displaying a time-dependent impact of increased NFX1-123 expression with 16E6. While this rise in MCM4 protein was not seen in HFK-A cells, there was greater expression of MCM4 in late 16E6/FN123 when compared to 16E6/vec cells (Fig. 9A, bottom Western blots). Higher expression levels of MCM2 and MCM4 in the late 16E6/FN123 relative to the VEC/vec cells across HFK biological backgrounds validated our HFK-C mass spectrometry findings and provided a potential direct link of the 16E6 and NFX1-123 partnership to increased levels of these proteins longitudinally.

## DISCUSSION

16E6 plays a significant role in inducing and maintaining cellular transformation of its infected host cell, despite having no enzymatic function (7, 12), so it must partner with host cell proteins to affect cellular dysregulation. Here we describe the role of NFX1-123 with 16E6 over time that induces longitudinal phenotypic growth changes and, with that, protein profile shifts.

NFX1-123 is increased in HPV 16-positive cervical cancers compared to normal tissues; NFX1-123 binds directly to 16E6 (19, 21); and greater NFX1-123 expression with 16E6 augments hTERT expression and telomerase activity (19, 21, 34). We have now demonstrated that, over time, 16E6 and increased levels of NFX1-123 impact proteins involved in the telomerase enzyme’s localization and function in the nucleus as well as in cellular DNA repair and replication. All of these are critical to the oncogenesis of HPV-associated cancers.

Although mass spectrometry did not detect the hTERT protein, HSP90 was upregulated in the late 16E6/FN123 compared to late 16E6/vec cells, and it is known that HSP90 is required for proper folding of the hTERT protein, a necessary step for its nuclear localization (26–30). Inside the nucleus, telomerase must be localized to the Cajal body, which is a specialized nuclear compartment enriched with macromolecules, including the telomerase RNA (30–32). The tailless complex polypeptide 1-ring complex/chaperonin containing tailless complex polypeptide 1 (TRiC/CCT) is a molecular chaperone that consists of eight proteins (CCT1–CCT8) which indirectly assist localizing the hTERT to the Cajal body (30, 33). All proteins in the TRiC/CCT family were upregulated in the late 16E6/FN123 compared to the late 16E6/vec cells. Increased proteins related to telomerase function and localization, as well as telomere regulation pathways, support our previously published findings that hTERT is influenced by the 16E6 and NFX1-123 partnership (19, 21) and give new insight into how the 16E6 and NFX1-123 partnership influences the telomerase complex beyond expression of its catalytic subunit, hTERT.

MCM proteins are a crucial component of the DNA replication and are a marker of cellular proliferation (35). This study is the first to demonstrate that NFX1-123 expression, over time, is associated with elevated MCM2 and MCM4. These proteins are known to be involved in several types of cancers, including cervical cancer (36–39). Although we saw MCM2 and MCM4 increased in our late 16E6/vec relative to its early time point, we know that NFX1-123 is an endogenous protein that remains present in 16E6/vec cells at late passages. However, with sustained, greater expression of NFX1-123, there was exaggerated increase in these MCM proteins over time, which matched clinical increases in MCM proteins seen in HPV-associated cervical cancers (37). Understanding the causal relationship between greater NFX1-123 expression and modulated upregulation of MCM proteins is important, as it informs whether a reduction in NFX1-123 expression would be a therapeutic target in HPV-associated disease states by broadly affecting MCM genes and its cellular pathways. Most interestingly, while the telomere regulation and localization pathways and DNA repair and replication pathways may appear to be distinct, we identified that these pathways share proteins and protein partnerships.

This study also lays the groundwork to determine how 16E6 and NFX1-123 collaboratively impact the expression of cellular proteins over time in infected cells, and future studies will parse out how these protein expression changes occur. NFX1-123 contains protein motifs that define it as a direct and indirect RNA regulatory protein as well as an E3-ubiquitin ligase (40); as such, we propose that NFX1-123 may be involved with both post-transcriptional and post-translational regulation of target gene pathways. The PAM2 motif of NFX1-123 is required to bind cytoplasmic poly(A) binding proteins (PABPCs). PABPCs bind to the poly(A) tail of mRNAs and help stabilize the transcription and recruit translational machinery on the mRNA to increase protein production (41); NFX1-123 requires this motif to increase hTERT and Notch1 expression (19, 34, 42). NFX1-123 also has an R3H domain which putatively binds to single-stranded nucleic acids, making NFX1-123 a protein that can bind RNA directly; this motif is also required to increase hTERT and Notch1 expression (19, 34, 42). Last, NFX1-123 has a RING domain, and RING domains have been shown to have E3 ubiquitin ligase functions, covalently attaching ubiquitin to target proteins, marking them for proteasomal degradation (43). We will determine whether these protein motifs found in NFX1-123 remain critical for the increased gene expression seen by proteome profile and Western blot over time and affect cellular growth and longevity.

This study reinforces the synergistic actions of HPV16E6 and NFX1-123 and identifies cellular pathways impacted by this protein partnership over time. These findings encourage further investigation of NFX1-123 as a potential therapeutic target of HPV 16-positive tissues.

## MATERIALS AND METHODS

### Cell culture

Primary HFKs were isolated from neonatal circumcision tissue by incubating in 7.5 mg/mL dispase (Thermo Fisher Scientific, 17105041) for 72 h at 4°C using procedures previously described (42). HFKs from three anonymous donors were utilized to obtain three biological replicates (HFK-A, HFK-B, and HFK-C).

### Virus production and transduction

Retroviruses were produced in 293T cells by a transient vesicular stomatitis virus G-pseudotyped production protocol previously described (44). After incubating the viral plasmids and plasmids of interest with FuGENE6 (Promega, E2692) in 293T cells, the viral supernatants were collected over 48 h. For transduction, viral stocks were concentrated by ultracentrifugation, mixed with polybrene (10 µg/mL), and incubated with 70% confluent HFKs for 3 hours. After incubation, the cells fed with keratinocyte growth medium (Promocell, C-20011). Then, 48 h following transduction, cells were placed under antibiotic selection. Cells transduced with 16E6 or empty vector pBABE pure underwent puromycin selection (0.5 µg/mL), after which cells were transduced with FN123 or empty vector LXSN and then underwent G418 selection (50 µg/mL). After selection was completed and confirmed by a non-transduced control HFK plate, media without antibiotic were used.

### Monolayer HFK growth assay

Cells were plated at a density of 500,000 cells/plate and counted every 3 days to calculate population doublings. Cell counting was performed using 0.01% trypan blue staining with the Bio-Rad Automated Cell Counter.

### Reverse transcription quantitative PCR

Cell pellets were lysed in TRIzol Reagent (Thermo Fisher Scientific, 15596026), and RNA was purified according to manufacturer instructions. SuperScript IV VILO Master Mix with ezDNase enzyme (Thermo Fisher Scientific, 11766050) was used to generate cDNA. Using a 1:60 dilution of the cDNA, quantitative RT-PCR was performed with the QuantStudio3 Real-Time PCR System (Thermo Fisher Scientific, A28131) with the PowerUp SYBR Green Master Mix (ThermoFisher Scientific, A25742). Primers for 16E6 (F: GCACAGAGCTGCAAACAACTATACA and R: TCCCGAAAAGCAAAGTCATATACC), NFX1-123 (F: CCACAGCTTCCCTCCCA, and R: CCTGGACGTCAAAATAGTCAA), FLAG-tagged NFX1-123 (F: GGACTACAAAGACGACGA and R: TGCCAAGGTTGATTCTGAA) and 36B4 (F: TGCCAGTGTCTGTCTGCAGA and R: ACAAAGGCAGATGGATCAGC) were used. Each sample was assayed in technical triplicates, and relative standard curves were generated for each target. Statistical significance was calculated using an unpaired, parametric, two-tailed *t*-test in GraphPad Prism version 10.4.1.

### Immunoblotting

Whole cell lysates were lysed in WE16 buffer (250 mM NaCl, 50 mM Tris-HCl, pH 7.5, 5 mM EDTA, 0.1% SDS, 01% NP40, 20% glycerol, 0.5 mM Na orthovanadate, 80 mM b-glycerophosphate, and 50 mM NaF) with cOmplete Mini EDTA-free Protease Inhibitor Cocktail (Roche, 11836153001). Lysates were quantified with the Bio-Rad DC Protein Assay (Bio-Rad, 5000112). Thirty microgram lysates were separated by gel electrophoresis in 4%–15% Tris-Tricine TGX gels and transferred to 0.2 µM polyvinylidene difluoride membranes using the Trans-Blot Turbo Transfer System (Bio-Rad, 4561084, 1704150). Primary antibodies used for protein detection were NFX1-123 (1:5,000; custom-made by Fortis Life Sciences to peptide sequence SNLQKITKEPIIDYFDVQD), p53 (1:5,000; Cell Signaling, 18032), MCM2 (1:5,000; Abcam, ab4661), MCM4 (1:1,000; Cell Signaling, 3228S), and GAPDH (1:100,000; Abcam, ab8245). Secondary antibodies were antimouse horseradish peroxidase (HRP) (1:5,000–1:100,000; Cell Signaling, 7076S) and antirabbit HRP (1:5,000-1:10,000, Cell Signaling, 7074S). Blots were visualized with SuperSignal West Pico PLUS Chemiluminescent Substrate (Thermo Fisher Scientific, 34580) on the ChemiDoc Imaging System. Densitometry was calculated in ImageJ by measuring integrated density and normalized to GAPDH.

### Organotypic rafting

Organotypic rafting of late passage HFK-C cells was performed as previously published (45). Rafts were fixed for 24 h at room temperature by immersion in 10% neutral buffered formalin. The specimens were then rinsed in distilled water and stored in 70% ethanol. Rafts were dehydrated through a graded series of ethanol, then cleared in xylene and infiltrated with three changes of paraffin (under vacuum at 59°C, 60 min each). Hematoxylin and eosin staining was performed using the Gemini AS automatic stainer.

### TRAPeze assay

Cellular extracts were obtained by lysing the cells with 50–100 µL of 1× CHAPS buffer containing 200 U/mL RNase inhibitor, and 100 ng protein extracts were used for TRAPeze Telomerase detection kit assays (Millipore, S7700) following the manufacturer’s protocol. The PCR-amplified product (24 µL) was run on a 20% polyacrylamide gel using acrylamide/bis-acrylamide 40% solution 19:1 (Fisher Scientific) for the Variable Comb Vertical system. The gel was stained with ethidium bromide (Fisher Scientific), and images were collected using the ChemiDoc Imager. Density of telomerase activity PCR product bands was determined using ImageJ analysis, and percent telomerase activity was calculated relative to control cells set as 100% activity as previously published (34).

### TMT sample preparation

Sample preparation was performed as in previously published protocols (46, 47). HFK-C cell pellets from early and late passages were resuspended in 100 µL 8 M urea and 100 mM Tris, pH 8.5. The resuspended cell pellet slurries were transferred to Bioruptor tubes (Diagenode, #0010010). Cells were lysed via Diagenode Bioruptor, 30 s on/30 s off, for 30 cycles. Samples were clarified by centrifuging for 30 min at 12,000 rcf. Supernatants were analyzed in a Bradford assay (Bio-Rad, 5000002) to determine protein concentration. Fifty micrograms of each sample was treated with 5 mM Tris (2-carboxyethyl) phosphine hydrochloride (Sigma-Aldrich, C4706) to reduce disulfide bonds, and the resulting free cysteine thiols were alkylated with 10 mM chloroacetamide (Sigma-Aldrich, C0267). Samples were diluted with 50 mM Tris-HCl, pH 8.5 (Sigma-Aldrich, 10812846001), to a final urea concentration of 2 M for overnight trypsin/Lys-C digestion at 35°C (1:50 protease:substrate ratio, Promega, V5072).

### Peptide purification and labeling

Proteolytic digestions were quenched with trifluoroacetic acid (TFA, 0.5% vol/vol), and peptide purification and labeling were performed following previously published procedures (46).

### High pH basic fractionation

One-fourth of the combined mixed, labeled peptides was resuspended in 0.5% TFA and fractionated on a Waters Sep-Pak Vac cartridge (Waters, WAT054955) with a 1 mL wash of water, 1 mL wash of 5% acetonitrile, 0.1% triethylamine (TEA) followed by elution in 12.5%, 15.0%, 17.5%, 20.0%, 22.5%, 25.0%, 30.0%, and 70.0% acetonitrile, all with 0.1% TEA. Eluted fractions were dried and then resuspended in 0.1% formic acid.

### Nanoflow liquid chromatography-tandem mass spectrometry

Nanoflow liquid chromatography-tandem mass spectrometry analysis was performed utilizing an EASY-nLC 1200 HPLC system (SCR: 014993, Thermo Fisher Scientific) coupled to Exploris 480 mass spectrometer with FAIMSpro interface run on a 25 cm Aurora Ultimate TS column (Ion Opticks, AUR3-25075C18-TS) in a 45°C column oven with a 180 min gradient. For each fraction, 25% of the sample was loaded and run at 350 nL/min with a gradient of 8%–38% B over 98 min; 30%–80% B over 10 min; held at 80% for 2 min; and dropping from 80% to 4% B over the final 5 min (mobile phase A: 0.1% formic acid (FA), water; mobile phase B: 0.1% FA, 80% acetonitrile (Thermo Fisher Scientific, LS122500). The mass spectrometer was operated in positive ion mode, default charge state of 2, advanced peak determination set to on, and lock mass of 445.12003 to ensure mass accuracy. Three field asymmetric ion mobility spectrometry compensation voltages (CVs) were utilized (−45 CV, −55 CV, and −65 CV) each with a cycle time of 2 s and with identical MS1 and MS2 parameters. Precursor scans (*m*/*z* 375–1,500) were done with an Orbitrap resolution of 60,000, RF lens percent set to 40, 50 ms maximum inject time, 300% automatic gain control (AGC) target, minimum MS2 intensity threshold of 5e4, MIPS mode set to peptide, including charges of two to eight for fragmentations with a 60 s dynamic exclusion setting. MS2 scans were performed with a quadrupole isolation window of 0.7 *m*/*z*, 32% higher-energy collisional dissociation collision energy, 45,000 resolution, 200% AGC target, 120 ms maximum IT, and a fixed first mass of 100 *m*/*z*.

### Mass spectrometry raw data analysis

Resulting RAW files were analyzed in Proteome Discover 2.5 (Thermo Fisher Scientific [48]) with a Homo sapiens UniProt reference proteome FASTA (downloaded 13 May 2022, total of 78,806 protein sequences) plus common laboratory contaminants (73 protein sequences) and the HPV16E6 protein sequence. SEQUEST HT searches were conducted with full trypsin digest, three maximum number missed cleavages, precursor mass tolerance of 10 ppm, and a fragment mass tolerance of 0.02 Da. Static modifications used for the search were (i) carbamidomethylation on cysteine (C) residues and (ii) TMTpro label lysine (K). Dynamic modifications used for the search were TMTpro label on peptide N-termini, oxidation on methionine (M) residues, phosphorylation on serine, threonine, and tyrosine (S, T, Y) residues, deamidation on asparagine and arginine (N and Q), methionine loss, or acetylation with methionine loss on protein N-termini. Percolator false discovery rate was set to a strict setting of 0.01 and a relaxed setting of 0.05. IMP-ptm-RS node was used for all modification site localization scores. Values from both unique and razor peptides were used for quantification. In the consensus workflows, peptides were normalized by total peptide amount with no scaling. Quantification methods utilized TMTpro isotopic impurity levels available from Thermo Fisher Scientific. Reporter ion quantification was allowed with an S/N threshold of 6 and co-isolation threshold of 30%. Resulting grouped abundance values for each sample type, abundance ratio values, and respective *P* values (ANOVA individual protein based) were exported to Microsoft Excel. Pathway analysis was performed using STRING version 12 (22).

## Data Availability

Raw and processed mass spectrometry data have been uploaded to the ProteomeXchange consortium member MassIVE repository (ftp://massive-ftp.ucsd.edu/v09/MSV000097715/) with accession number MSV000097715 and cross referenced to ProteomeXchange as PXD063286.
